# Editorial: The immune infiltrate as a paradigm model to study the biology and novel therapeutic approaches in sarcomas, volume II

**DOI:** 10.3389/fendo.2025.1619528

**Published:** 2025-05-19

**Authors:** Silvia Vanni, Federica Recine, Lorenzo D’Ambrosio, Robin Lewis Jones, Alessandro De Vita

**Affiliations:** ^1^ Preclinic and Osteoncology Unit, Biosciences Laboratory, IRCCS Istituto Romagnolo per lo Studio dei Tumori (IRST) “Dino Amadori”, Meldola, Italy; ^2^ Medical Oncology Unit, Azienda Ospedaliera “San Giovanni Addolorata”, Roma, Italy; ^3^ Department of Oncology, University of Torino, Torino, Italy; ^4^ San Luigi Gonzaga Hospital, Orbassano, Italy; ^5^ Royal Marsden Hospital and Institute of Cancer Research, London, United Kingdom

**Keywords:** immune infiltrates, sarcomas, tumor microenvironment, TME, bone sarcoma, soft tissue sarcoma

Sarcomas, a heterogeneous group of malignant tumors originating in bone and soft tissues, continue to challenge oncologists with their rarity, heterogeneity, and resistance to conventional therapies. While significant progress has been made in molecular characterization, immune profiling, and targeted therapies, many gaps remain in our understanding of these tumors’ behavior and treatment response (1). This Research Topic brings together six studies that represent a cross-section of contemporary sarcoma research-from mechanistic insights and prognostic modeling to innovative case reports and novel therapeutic strategies.

In a compelling contribution to the field of nanomedicine (2), Cai and He demonstrate that exosomes derived from bone marrow mesenchymal stem cells (BMSCs) can serve as highly effective vectors for doxorubicin delivery in osteosarcoma treatment. By engineering hybrid exosomes (HEs) through fusion with liposomes, the authors achieved a drug delivery system that exhibited superior tumor-targeting ability, pH-sensitive drug release, and enhanced cytotoxicity in both cell line and mouse models. These findings underscore the potential of BMSC-derived exosomes to revolutionize osteosarcoma therapy through precision-targeted nanocarriers with minimal systemic toxicity.

Complementing this innovative therapeutic approach is a rare and insightful clinical observation by Bolukbas et al., who report a spontaneous, systemic anti-tumor response -known as the abscopal effect **-** in a patient with recurrent undifferentiated pleomorphic sarcoma. Following localized radiotherapy, the patient experienced a complete regression of distant metastases without additional systemic treatment. This case adds to the small but growing body of literature documenting the immunogenic potential of radiotherapy in sarcoma, traditionally considered an “immune-cold” malignancy (3). It also invites deeper exploration of the conditions under which T-cell activation and systemic immune responses might be harnessed in sarcoma care.

Building on the intersection of immunity and tumor progression (4), Huang et al. present a robust immune-related prognostic model for synovial sarcoma, integrating gene expression data to identify biomarkers associated with poor survival and potential metastatic behavior. This model offers not only prognostic value but also mechanistic clues, pointing to immune pathways that could serve as future therapeutic targets in this rare and aggressive sarcoma subtype.

The importance of precise prognostication is echoed in Han et al.’s study, which introduces a nomogram model for metastatic soft tissue sarcoma survival. By incorporating clinical variables such as tumor size, histologic type, and metastasis pattern, this tool enhances risk stratification and may support more personalized treatment planning. As soft tissue sarcomas encompass over 100 histological subtypes, such models are vital for individualized care and decision-making in metastatic settings.

In another important contribution to the landscape of sarcoma diagnosis, Huang et al. investigate PITX1 as a novel marker for grading and immune infiltration in chondrosarcoma. Through integrative analysis of public datasets and immunohistochemical validation, the study reveals that PITX1 expression correlates with tumor grade and immune cell infiltration - suggesting a potential dual role as a prognostic biomarker and a modulator of the tumor immune microenvironment. These findings pave the way for immunologically informed classification systems and may inform future immunotherapy approaches (5).

Rounding out the Research Topic is a rare case report by Chen et al., who describe the development of perianal leiomyosarcoma as a late sequela of pelvic radiotherapy for rectal cancer. The report provides a cautionary lens on long-term surveillance and secondary malignancy risk in previously irradiated fields. While such cases are exceedingly uncommon, they remind clinicians of the need for vigilance in survivorship care, especially in light of growing long-term cancer survivorship.

Taken together, the studies in this Research Topic reflect a multi-dimensional approach to understanding and managing sarcomas. Several common threads emerge: the growing utility of molecular and immunological markers for prognosis and therapy, the potential of nanotechnology and engineered biologics in drug delivery, and the vital contributions of rare clinical cases in expanding our therapeutic horizons.

Moreover, the diverse methodologies employed - from bioinformatics modeling and preclinical *in vivo* validation to immunohistochemistry and case-based narrative -illustrate the importance of cross-disciplinary collaboration in addressing the unique complexities of sarcoma.

Looking ahead, these studies invite further exploration of:

- Immune system manipulation as both a therapeutic and diagnostic tool in sarcoma;
- Biomimetic delivery systems capable of overcoming physiological barriers and drug resistance;
- Long-term implications of treatment modalities, particularly radiotherapy, in secondary tumor development;
- Data-driven approaches to precision oncology that account for the rare and variable nature of sarcomas.

In conclusion, this Research Topic represents a valuable contribution to the evolving field of sarcoma research. By integrating innovations in immunology, targeted therapy, prognostication, and clinical observation, these six studies provide a strong foundation for future discoveries that can inform both bench-side investigations and bedside interventions.
